# "Intelligent" descriptions of microbial kinetics in finitely dispersed bioreactors: neural and cybernetic models for PHB biosynthesis by *Ralstonia eutropha*

**DOI:** 10.1186/1475-2859-6-23

**Published:** 2007-08-08

**Authors:** Pratap R Patnaik

**Affiliations:** 1 Institute of Microbial Technology, Sector 39-A, Chandigarh-160036, India

## Abstract

**Background:**

For many microbial processes, the complexity of the metabolisms and the responses to transient and realistic conditions are difficult to capture in mechanistic models. The cells seem to have an innate intelligence that enables them to respond optimally to environmental changes. Some "intelligent" models have therefore been proposed and compared with a mechanistic model for fed-batch cultures of *Ralstonia eutropha*.

**Results:**

Two kinds of models have been proposed to describe such cellular behavior. Cybernetic models are derived through postulates of cellular intelligence and memory, and neural models use artificial intelligence through neural networks. Some competing models of both kinds have been compared for their ability to portray and optimize the synthesis of poly-β-hydroxybutyrate by *Ralstonia eutropha *in fed-batch cultures with finite dispersion. Neural models enabled the formation of more of the polymer than cybernetic models, with lesser utilization of the carbon and nitrogen substrates. Both types of models were decidedly superior to a mechanistic model used as a reference, thus supporting the value of intelligent descriptions of microbial kinetics in incompletely dispersed bioreactors.

**Conclusion:**

Neural and cybernetic models describe and optimize unsteady state fed-batch microbial reactors with finite dispersion more effectively than mechanistic models. However, these "intelligent" models too have weaknesses, and hence a hybrid approach combining such models with some mechanistic features is suggested.

## Background

Microbial growth, substrate utilization and product formation in bioreactors have traditionally been described by algebraic or differential equations derived on the principles of chemical reactions. These so-called mechanistic models are adequate for gross descriptions of culture behavior in small (laboratory-scale) cultivation vessels. Such bioreactors are usually operated under largely "ideal" conditions, implying that the fermentation broth is homogeneous, there are no disturbances, there is approximately balanced growth, and data acquisition and control systems are sufficiently elaborate, fast and accurate.

These ideal conditions do not, however, prevail in the more "real" situations of pilot-scale and production-scale bioreactors. These larger reactors have spatial variations within the vessel, influx of noise from the environment, and restricted use of monitoring and control devices because of practical and financial considerations [1,2]. Culture behavior in such "nonideal" situations is often quite different from that in ideal reactors, and mechanistic mathematical models developed on the basis of laboratory-scale observations become inapplicable or too approximate or may require frequent adjustments of the parameters during the fermentation process [3,4].

Ideal descriptions sometimes do not apply even to small-scale cultures, especially when these are sensitive to fluctuations in the extra-cellular environment and/or complex metabolic processes underlie the macroscopically observed behavior. The synthesis of the aspartate family of amino acids in *Corynebacterium lactofermentum *[5], cephalosporin C production by *Cephalosporium acremonium *[6], and even relatively robust processes such as acetic acid production [7] and the biosynthesis of poly-β-hydroxybutyrate [8] illustrate the limitations of the classical chemical kinetic approach to the modeling of complex microbial kinetics.

Recognition of such limitations has led to recent proposals for "intelligent" descriptions of microbial cultures in nonideal bioreactors [9-11]. These are broadly of two kinds. One kind of description ascribes to living cells the ability to think, remember and act accordingly. The cells are thus considered to have some rudimentary intelligence similar to those of higher organisms. This approach has led to the class of cybernetic models discussed in recent reviews [11,12]. The second approach utilizes methods of artificial intelligence (AI) to describe quantitatively the observed behavior of cellular systems and predict their performance under different conditions. Commonly used AI techniques include artificial neural networks, fuzzy logic, expert systems and genetic algorithms, often in combination with some mathematical equations [13].

The availability of two different streams of intelligent modeling, and the continuing use of classical mathematical models, poses the question of which approach to adopt in a given application. Since there are few comparative studies of intelligent models vis-à-vis mechanistic models, there is yet no general answer. The present study provides more information to help formulate guidelines to choose one or more modeling approaches in a given situation. The microbial process investigated is the synthesis of poly-β-hydroxybutyrate (PHB) in fed-batch cultures of *Ralstonia eutropha*. The reasons for choosing this fermentation and a brief description of it are provided next.

## Biosynthesis of PHB

PHB is an energy-storage polymer that is synthesized by some bacteria under conditions unfavorable to their growth. It is commercially important because of its similarities with competing polymers such as polyethylene (PE) and polypropylene (PP) that are produced on a large scale. PHB can replace PE and PP because, as a copolymer with polyhydroxyvalerate and similar polymers, PHB has many properties comparable to those of PE and PP. In addition, whereas PE and PP are synthesized from petroleum sources at high temperature and pressure, PHB can be produced from renewable resources by microbes under milder and less energy-consuming conditions.

Petroleum-based chemically synthesized polymers also present environmental problems since they are difficult to degrade; by contrast, PHB is biodegradable and biocompatible. While the similarity of its properties to those of PE and PP enable PHB to be used to be used for similar applications, its compatibility with body tissues widens its potential uses to medical areas such as surgical sutures, wound dressings and ocular devices. Recent reviews [14-16] have discussed these aspects in detail.

In spite of its decisive benefits, industrial production of PHB is still behind those of PE, PP and other polyalkenes. Raw material costs and energy consumption obviously cannot explain this lag. A major reason is the low productivity of fermentations for PHB. This is due to the inadequate and imprecise modeling and optimization of PHB fermentations under industrially relevant conditions. Whereas conventional modeling methods work satisfactorily for small bioreactors, they have limited validity for large reactors, where the behavior of both the microbial culture and the reactor are often significantly different [15-17]. Such situations requite intelligent models that can capture subtle variations and function through cumulative knowledge of culture behavior.

*Ralstonia eutropha *(earlier called *Alcaligenes eutrophus *and recently renamed *Cupriavidus necator*) is possibly the most widely used organism for PHB production. The generation of high concentrations of PHB requires good growth of the cells and a method to induce them to synthesize the polymer [18,19]. Growth requires adequate supply of a carbon source, which is usually fructose or glucose. Since *R*. *eutropha *synthesizes PHB (intra-cellularly) under adverse growth conditions, in a bioreactor the cells are subjected to stress by depriving them of nitrogen [20] or phosphorus [21], the former being more commonly employed. Nitrogen concentration in the bioreactor is usually controlled by regulating the supply of ammonium chloride or sulfate. Although a shortage of nitrogen induces the cells to synthesize PHB, excessive deprivation retards cell growth [22] and causes depolymerization of PHB [23,24]. Both factors contribute to a lowering of the overall concentration of PHB in the fermentation broth.

Biomass growth obviously requires an adequate supply of carbon. However, excess of carbon also inhibits growth [22]. These observations and the metabolic network for PHB formation [16] indicate that control of the rates of inflow of nitrogen and carbon is both complex and critical for high production efficiencies. Therefore, fed-batch fermentation is the preferred mode of operation. However, the optimum time-dependent flow rates seem to vary greatly among different studies, depending on the kinetic model employed and the optimization strategy.

Optimization of the fermentation depends on quantitative modeling of its kinetics. Mechanistic models are the oldest, still in use but slowly being replaced, at least partially, by intelligence-based models. In this study the well-established mechanistic model of Lee et al. [25] was used as a reference to compare with three cybernetic models and a number of neural network models. These are introduced next, followed by a description of the data generation method for a simulated nonideal bioreactor.

## Introduction of the kinetic models

Recent studies by this author [8,26] have been based on a mechanistic model formulated by Lee et al. [25], who monitored the concentrations of four state variables in a fed-batch culture of *R. eutropha*: PHB, residual biomass, glucose (the carbon source) and ammonium chloride (the nitrogen source). The residual biomass (hereafter called the biomass for conciseness) is the difference between the total mass of cells and their PHB content. Given the (time-dependent) feed rates of the two principal substrates, glucose and ammonium chloride, differential mass balances may be written for the measured concentrations. A salient feature of the model was that the specific rates for biomass growth and polymer (PHB) synthesis included the observations that (a) viable cells can generate some PHB without ammonium and (b) high intra-cellular concentrations of the polymer inhibit cell growth [22,27]. The optimized performance of their bioreactor is compared here with those described by the intelligent models.

Mechanistic models have a chemical reaction framework that imparts simplicity but ignores regulatory processes within the cells and restricts their flexibility to adapt to dynamic conditions, where disturbances and nonhomogeneity may be variable and significant events. Therefore, such models are wanting in their ability to portray lag phase behavior, diauxic and triauxic growth, and the transient responses that follow perturbations to continuous and fed-batch cultures. Dhurjati et al. [28] proposed the cybernetic approach as an alternative. Their basic tenet was that living cells possessed an innate "intelligence", whereby they could adjust their internal metabolism and the resulting responses so as to maximize their survival under varying conditions. This evolutionary concept was formally expressed by maximizing an objective function such as the growth rate.

Yoo and Kim's [29] cybernetic model for PHB has been the forerunner for two other models, all of which have been evaluated in this study. Like Lee et al. [25], they divided each cell into two components: residual biomass and PHB. A key assumption was that the cells allocate the carbon source to the enzyme synthesis system such that at all times they have considerable catabolic flexibility under nitrogen starvation. This aspect is discussed later.

The original cybernetic formalism [28] is based on Herrnstein's matching law [30], which requires the fractional allocation of resources to a set of activities to match the fractional returns. Since this approach resulted in stiff differential equations, Yoo and Kim [29] modified it to a nonsingular optimal strategy that maximized the cell mass at each instant of time. Ferraz and coworkers [18] expanded the model to include different enzymatic induction and repression strategies, and cells with different morphological features. The complete equations are available in their paper, and are not reproduced here. Gadkar et al. [31] presented a metabolically more structured version of Yoo and Kim's [29] model, while retaining the basic idea of dividing each cells conceptually (a) residual biomass and (b) PHB. In addition, the reactor mass balances included a lumped concentration of the internal metabolites. Gadkar et al. also incorporated Belfares et al's [32] observation that at high concentrations the biomass itself becomes self-inhibitory, and they proposed global competition between glucose uptake and the degradation of PHB.

To be able to express all the relevant features of a microbial system under different conditions, cybernetic models tend to be quite complex. For instance, Ferraz et al.'s [18] model has 53 parameters and 11 dependent variables. Two other difficulties with these models are: (a) the inability to establish a correspondence between the key enzymes in a model and the enzymes in the actual metabolic network and (b) the possibility of more than one cybernetic goal meeting the desired objective equally well.

**Figure 1:**
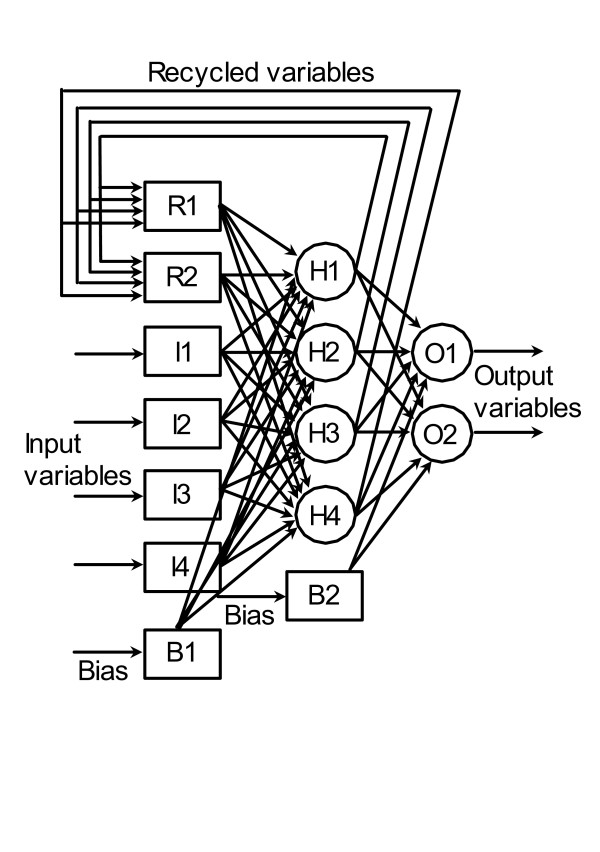
Schematic diagram of a typical neural network. I1 – I4 = input neurons; H1 – H4 = hidden neurons; O1, O2 = output neurons; B1, B2 = bias neurons; R1, R2 = recurrent neurons. The numbers of hidden, recurrent and bias neurons are adjustable.

Neural networks provide an alternative. Like cybernetic models, they have a cognitive approach. By learning directly from the performance of a culture, neural networks evolve internal structures and information cycles that enable them to mimic a real process. Cellular intelligence is ingrained in the architecture of a neural network through the arrangement of information processing elements called neurons. The name 'neuron' and the flow of information among neurons are intended to mimic the functioning of biological neurons. Neurons in a network are arranged typically into an input layer, an output layer and one or two intermediate layers (called hidden layers). Figure 1 depicts a network with four neurons each in the input and hidden layers and two each in the other layers. The recurrent neurons (R1 and R2) are present only in some types of networks (the Elman and Hopfield types in this study) and the bias neurons (B1 and B2) are optional. Networks differ in the manner in which the neurons are laid out, their processing functions, and the directions of the information flow streams.

Since many different configurations of neural networks are possible, it is important to be able to sift useful configurations from ineffective ones at an early stage of application. As an aid to this, Patnaik [33] developed a library of networks used commonly for microbial processes; inherent in this library was a set of rules to screen competing networks and select a few promising ones for detailed studies. Both the library and the screening rules may be updated. Its current version was applied recently [8] to a fed-batch culture of *R. eutropha *to maximize PHB formation at optimum dispersion. Dispersion is an important nonideal feature. Whereas the fluid in small bioreactors is usually fully dispersed, this possibility reduces with increasing size. It was shown recently [17] that PHB production is highest at a finite optimum dispersion. The application of a library of neural networks to such a bioreactor revealed that while a recurrent Elman network generated the highest concentration of PHB, a radial basis topology (either in stand-alone mode or with generalized regression) provided faster optimization with slightly inferior results. These three neural network models were therefore chosen for the present analysis.

## Data generation

Whereas the broth is sensibly homogeneous in small laboratory-scale bioreactors, spatial variations become significant in the larger pilot- and production-scale vessels [3,34,35]. In other words, small bioreactors have nearly complete dispersion while large reactors do not. Achieving complete dispersion is also impractical in large fermentation vessels. Given this difficulty, it was shown in a recent simulation study [17] that dispersion corresponding to a Peclet number of Pe = 20 maximizes the production of PHB. The Peclet number is defined as:

Pe = uL/D_e_

Here u is the mean velocity of fluid flow in the bioreactor, L is a characteristic dimension of the vessel and D_e _is the effective dispersion coefficient. For small bioreactors, with nearly complete dispersion, D_e _→ ∞ and hence Pe → 0. The other extreme of D_e _→ 0 and Pe → ∞ corresponds to the total absence of dispersion, also referred to as plug flow. Real reactors have finite non-zero values of Pe.

Proprietary and commercial considerations often restrict the availability and disclosure of data from real industrial-scale fermentations. To overcome this difficulty, a common approach [36-38] is to generate data mimicking such a fermentation by adding nonideal features to a model that has been validated with laboratory-scale data. This approach was followed recently [8,26] for PHB biosynthesis by *R. eutropha*; the kinetic equations of Lee et al. [25] were inserted into the standard mass balance equations of a fed-batch bioreactor [39] and the model was solved with Pe set at the optimum value of 20 [17].

The plots of the concentrations of glucose, ammonium chloride, biomass and PHB were then sampled to obtain data representative of a nonideal (simulated) bioreactor. Although a uniform sampling interval is easy to implement, it is not desirable when there are large variations between different variables and with time for any particular variable. A fixed interval may then clutter the sample space with too many points from shallow regions while omitting important changes in sharply varying regions. As a result, the sampled data do not faithfully portray all the relevant features of the real process. One useful method to vary the sampling interval is to make it inversely proportional to the local gradient of the concentration profile. This generates closely spaced data when there are steep variations, while fewer data are sampled from mildly changing regions. This method was adopted here and earlier [40] to generate data simulating an optimally dispersed fed-batch fermentation.

## Results and discussion

**Figure 2:**
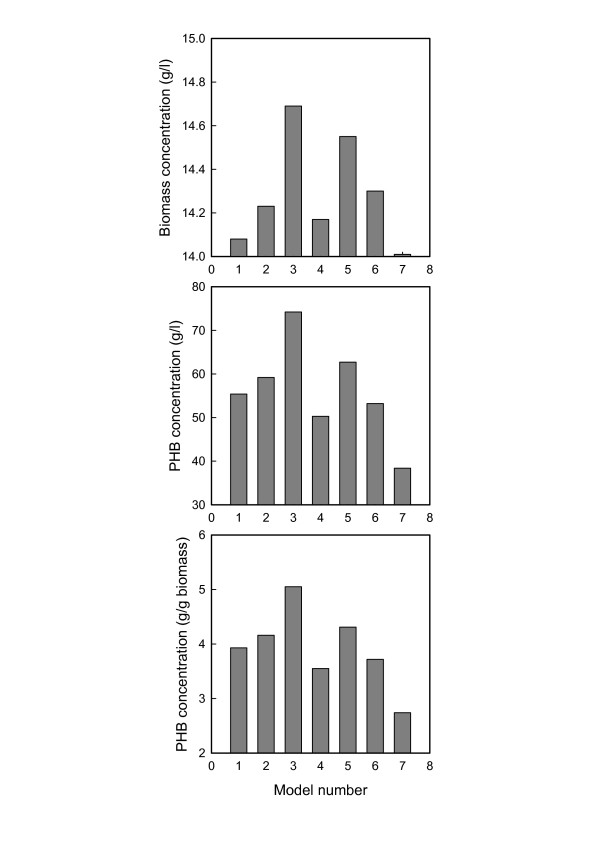
Peak concentrations of residual biomass and PHB achieved through different models. 1 – radial basis neural (this work); 2 – radial basis neural with generalized regression (this work); 3 – recurrent Elman (this work); 4 – cybernetic [29]; 5 – cybernetic [18]; 6 – cybernetic [31]; 7 – mechanistic [25].

Results from a recent analysis of this system [40] provided the starting point for the present study. That analysis compared the optimized results for a nonideal (Pe = 20) fed-batch bioreactor represented by each of seven neural networks: feed-forward with backpropagation (FFBP), FFBP with momentum (FFBPM), FFBP with adaptive learning (FFBPA), radial basis (RB), RB with generalized regression (RBG), Elman (ELM) and Hopfield (HOP). While the ELM configuration generated the highest concentration of PHB, the two radial basis versions resulted in slight lower outputs but converged faster to the optimum performance. Lyapunov exponents of the concentration profiles also showed that the RB and RBG networks produced a more stable fermentation than the Elman network. Both stability and speed of convergence are important for automated control of large bioreactors because variations in external conditions may require rapid responses to main high productivity. Therefore, all three neural models were selected for comparison with the three cybernetic models discussed before and the mechanistic model of Lee et al. [25]. The optimized final concentrations (at the end of 50 h of fermentation) for the biomass and PHB are compared in Fig. 2. While the Elman representation produces decisively higher concentrations, this is negated by much slower responses, whereas the RBG portrayal has a good balance of all the important features [40]. Figure 2 shows that the best cybernetic model (for this fermentation) is that of Ferraz et al. [18], and the performance it generates differs from that of the RBG neural model in a number of significant features. Whereas biomass growth according to Ferraz et al. is just 2.25% more than by neural optimization, PHB outputs are 1 1/2 to 2 times greater (5.91% in mg/l and 3.60% in g/g biomass). These differences are even more pronounced between each of these models and the mechanistic model of Lee et al. [25]. PHB production by the cybernetic and neural formalisms are 63.28% and 54.17%, respectively, larger for the volumetric concentration, with marginally lower improvements in terms of biomass concentration. On the contrary, the biomass itself does not increase comparably, being just 1.57% for the RBG neural network and 3.85% for Ferraz et al's [18] cybernetic description.

These results underline a fundamental advantage of "intelligent" models over mechanistic models. By being able to use information on cellular responses to derive reasoned inferences about the best operating conditions at any time, neural and cybernetic models manipulate the control strategies such that ammonium chloride and glucose supplied are channeled more into PHB synthesis pathways than into cell growth. Owing to its rigid chemical reaction approach, which ignores intra-cellular regulatory controls [12,28] and knowledge-based responses to evolving environmental conditions [11], the mechanistic approach is restricted in its ability and flexibility to allocate the available resources optimally to favor polymer formation more than cell growth [16].

**Figure 3:**
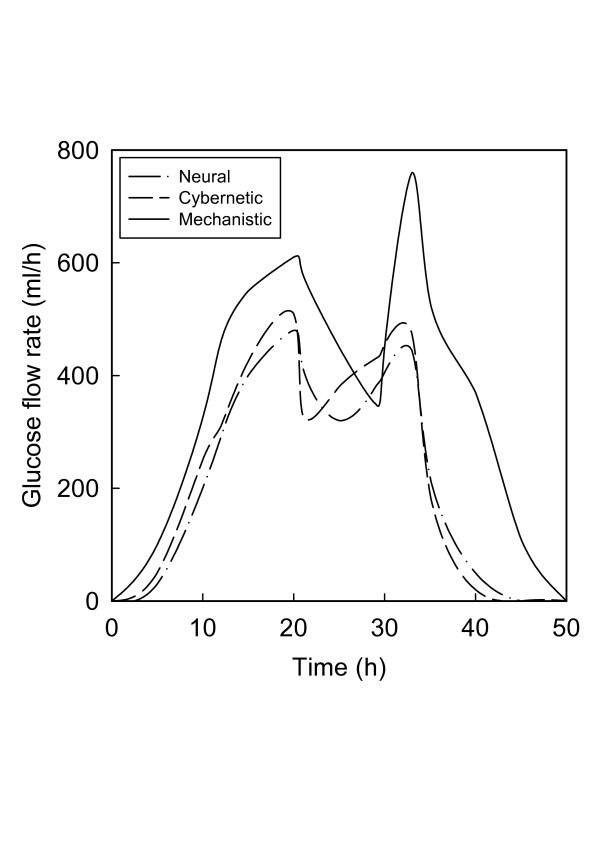
Optimal glucose feed rates according to the mechanistic model and the best cybernetic and neural models.

**Figure 4:**
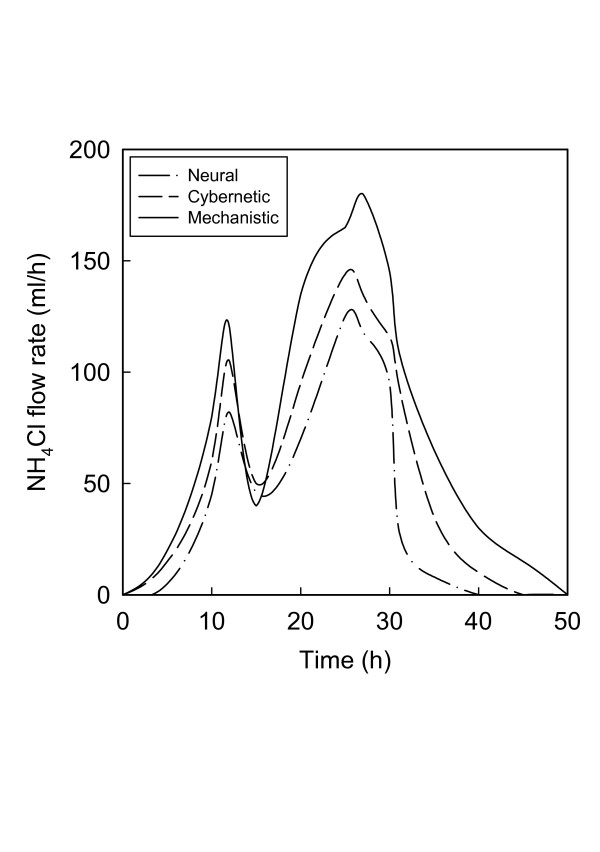
Optimal ammonium chloride feed rates according to the mechanistic model and the best cybernetic and neural models.

Besides the final concentrations shown in Fig. 2, similar differences are also seen in the time-variant feed rates of the two primary substrates. The inflow rates of both glucose (Fig. 3) and the ammonium salt (Fig. 4) are generally lowest for the RBG neural model and highest for the mechanistic model. The visual differences are confirmed by the actual quantities of substrates utilized (Table 1). The cybernetic approach is seen to be intermediate between the mechanistic and neural approaches, in terms of the actual quantities of the substrates as well as their ratio.

**Table 1:** Total consumption of substrates by different kinds of models to describe fed-batch biosynthesis of PHB by *Ralstonia eutropha*.

Type of model	Glucose (liters)	Amm. chloride (liters)	Ratio
Neural	10.250	1.924	0.188
Cybernetic	11.084	2.175	0.196
Mechanistic	17.451	3.680	0.211

The differences in the ratio of the nitrogen to carbon sources and the bimodal nature of the feed rate profiles may be interpreted in terms of the substrate distribution patterns in the metabolic network for PHB synthesis. While a detailed discussion of the metabolic system is not within the scope of this study, metabolic analyses of this process [41,42] indicate that in a heterogeneous broth both carbon and nitrogen supply have to be varied within certain ranges such that an optimum ratio is maintained all times. Too much of carbon and too little of nitrogen are detrimental to cell growth [22], while excessive shortage of either resource triggers depolymerization of PHB [23,24] since the cells then utilize PHB for their growth requirements. The optimum carbon: nitrogen ratio varies nonlinearly with time [8,18,31,38], further highlighting the complexity of interactions between cellular metabolism and dispersion in the broth. As discussed elsewhere [17], the degree of dispersion controls the rates of formation and consumption of acetate, a critical intermediate that exerts feedback control on the metabolic system [42,43]. Dispersion also plays a vital role in determining the balances between the synthesis and the degradation of PHB.

At high dispersion (Pe → 0), the nitrogen and carbon substrates may be freely accessed by the cells throughout the broth. The resultant acetate inhibits cell growth, thus reducing the volumetric productivity of PHB, even though its intra-cellular concentration may be high [44]. On the contrary, poor dispersion restricts the availability of the substrates, thus lowering the synthesis of PHB by the cells. Dispersion corresponding to Pe = 20 seems to provide the right balance between these opposing factors; given the complexities of the metabolic network and the utilization patterns of carbon, nitrogen and oxygen, intelligent models may be expected to learn the relevant features more accurately and reliably, and thus be able to exercise more effective control of the fermentation process. As the present results have shown (Fig. 2 and Table 1), this effectiveness is more for neural models than for cybernetic models, possibly because the former posses greater flexibility to adjust themselves with increasing knowledge of the process, and they are more robust to fluctuations both within [45] and outside [36,40] the microbial system.

## Concluding observations

This study has explored the relative merits of cybernetic, neural and mechanistic descriptions of microbial kinetics in terms of their ability to optimize PHB production by *R. eutropha *in fed-batch cultures. To mimic a large nonideal bioreactor the degree of dispersion of the fermentation broth was set at Pe = 20, shown earlier [17] to be the best value.

Under these conditions the best two neural network representations, viz. the Elman form and the radial basis network with generalized regression, generated higher concentrations of PHB than the best cybernetic model [18]. All these three models were superior to the mechanistic model of Lee et al. [25], used as a reference for data generation and comparison. The cybernetic and neural approaches also resulted in lower consumptions of the nitrogen and carbon substrates. One similarity, however, was that the feed rates of these substrates by all three approaches had bimodal distributions over the duration of the fermentation (50 h). The bimodality is consistent with the metabolic requirement that the substrate concentrations should vary within prescribed ranges for optimum flux distributions and to avoid growth inhibition and product degradation [22-24].

The superiority of intelligent approaches such as the neural and cybernetic methods under realistic conditions points both to the limitations of mechanistic models and to the complexity of cellular responses to environment changes. However, the cybernetic and neural methods also have both weaknesses and strengths, suggesting the possibility of developing intelligent hybrid descriptions that combine more than one kind of intelligent model with a segment of mechanistic modeling. Recent successes with hybrid neural models, i.e. combinations of mechanistic and neural models, indicate the feasibility of a hybrid neural-cybernetic-mechanistic approach for microbial kinetics. While this is the subject of future work, an exploratory analysis [46] has provided a road map for the development of such models.

## Competing interests

The author(s) declare that they have no competing interests.
